# Suppression of sphingosine 1-phosphate lyase retards the liver regeneration in mice after partial hepatectomy

**DOI:** 10.1042/BSR20200592

**Published:** 2020-07-10

**Authors:** Yuko Kageyama, Baasanjav Uranbileg, Yoshika Kusumoto, Eri Sakai, Hitoshi Ikeda, Makoto Kurano, Yutaka Yatomi

**Affiliations:** Department of Clinical Laboratory Medicine, The University of Tokyo, Tokyo, Japan

**Keywords:** Liver regeneration, S1P lyase, S1P

## Abstract

Background: Liver regeneration is an extremely complicated process that is regulated by a number of signaling pathways. Sphingosine 1-phosphate (S1P), a potent bioactive lipid mediator playing crucial roles in various cellular responses through its receptors, has been attracting attention in the fields of hepatology, where S1P lyase (SPL), an irreversibly degrading enzyme of S1P, reportedly has a stimulatory role in growth of hepatocellular carcinoma (HCC).

Aim of the study: To examine whether SPL might play a stimulatory role in liver regeneration.

Method: Using *in-vivo* siRNA technology, we inhibited SPL expression. Seventy percent of the liver was resected in mice as partial hepatectomy (PH). Liver tissue samples were collected and mRNA expression level of the SPL, IHC of the proliferating cell nuclear antigen (PCNA), protein levels of various proliferation factors and lipid measurements were performed in different groups.

Results: The mRNA levels of SPL increased in PH mice on the third day after PH surgery. When we suppressed the expression of SPL by *in-vivo* siRNA, we observed a significant decline of the PCNA positive cell numbers. Furthermore, the Cyclin D1 expressions and phosphorylation of ERK also were decreased in the siSPL injected PH group.

Conclusion: We verified the importance of the SPL in liver regeneration, using the mice PH model. SPL might be a potential target to facilitate liver regeneration.

## Introduction

The unique ability of the liver is to regenerate and regulate to keep its own size. The regeneration of the liver occurs after either surgical resection or injury-induced cell death. Scientific reports on liver regeneration appeared first in the late 19th century, and still remaining as an interesting and active area of the liver research [1]. The intense research to understand the mechanism that regulates the liver regeneration has revealed a number of details including cell cycle kinetics, signaling pathways in stimulating cell proliferation and humoral control of the liver regeneration [2–4]. The process of liver regeneration is driven by many factors including cytokines, chemokines, growth factors, and hormones [2,5–9]. Along with these factors, lipid mediators, such as sphingosine 1-phosphate (S1P), have been demonstrated to be involved in the liver regeneration [10,11]. S1P is a potent bioactive sphingolipid as extra- and intracellular regulator of growth, differentiation, migration, survival, angiogenesis, and other cellular responses through G protein-coupled cell surface receptors, as known as S1P_1–5_ [12–17]. Biosynthesis of S1P is through the phosphorylation of sphingosine by sphingosine kinase 1 and 2 (SK1, SK2) which are ubiquitously expressed in most tissues, SK1 expression was predominant in lung and spleen, whereas SK2 in liver and heart [18,19]. Degradation of the S1P bears by either S1P phosphatases (SPP) where de-phosphorylation occurs or S1P lyase (SPL), which facilitates irreversible degradation by yielding ethanolamine phosphate and hexadecenal as an intermediate in the sphingolipid to glycerophospholipid metabolic pathway [20]. The cellular level of S1P is controlled by the actions of the enzymes responsible for its formation and degradation. Regarding roles of S1P in the pathophysiology of the liver, we found that S1P has an inhibitory effect as an extracellular mediator on hepatocyte proliferation but has a stimulatory effect on the hepatic stellate cell proliferation which located in the Disse space, play an important role in wound healing on liver injury and possible subsequent fibrogenesis [21,22] and found possible involvement of the S1P in liver regeneration as an inhibitory effect through S1P_2_ receptor [23].

We also recently observed increased expression levels of the S1P-generating enzymes SKs together with an enhanced level of degrading enzyme SPL in human hepatocellular carcinoma (HCC) tissues [24]. *In-vitro* experiments showed that inhibition of SPL expression by siRNA led to reduced proliferation and invasion, while overexpression of SPL caused enhanced proliferation of HCC cell lines. We also reported a similar effect of the SPL on cell proliferation in human colorectal cancer cell lines [25]. Based on these evidences of the involvement of SPL in the proliferation of cell lines derived from HCC, we would predict possible participation of SPL in liver regeneration. To address this prediction, we used liver tissues from partial hepatectomy (PH) model mice which is known to be the most reliable model to study regeneration with minimal liver injury, synchronized cell cycle, and very high reproducibility with inhibition of the SPL.

## Materials and methods

### Animals

Male C57 BL/6J (8 weeks old, mean weight: 23.0 grams) mice (SLC, Shizuoka, Japan) were given a standard pellet diet and water housed in a 12-h light/12-h dark cycle *ad libitum*. We used three to ten mice for each experimental group. All the animal experiments were conducted in accordance with the guidelines for the Care and Use of Laboratory Animals and were approved by the ethic committee for the animal experiment of The University of Tokyo (approval number H17-125). The mice were anesthetized by an intraperitoneal injection of sodium pentobarbital (Somnopentyl, Kyoritsu Seiyaku Co., Tokyo, Japan) at 40 mg/kg body weight. After the sampling of liver tissue samples, the mice were sacrificed by cervical dislocation without recovery from anesthesia. All the animal experiments were conducted at an animal laboratory in the University of Tokyo. All the mice were maintained under specific pathogen-free conditions.

### Surgical procedure and experimental design

PH was performed as previously described [26]. To inhibit the expression levels of the SPL, we injected siSPL (*n*=10) or control (siNC) (*n*=8) into the tail vein of the mice and after 2 days when SPL is inhibited, we performed PH. At day 3 after surgery, liver tissue samples were collected for further analysis.

### *In-vivo* transfection of the SPL siRNA

For the RNA interference assays, *in vivo* pre-designed siRNA of the SPL was used (Ambion by Life Technologies, Thermo Fisher Scientific, Massachusetts, U.S.A.). Among three different siRNAs with distinct sequences were tested for SPL silencing and following siRNA selected for further usage. The sequence of SPL siRNA was sense- CAUUUUCGGUGAUCCUCAAtt, antisense- UUGAGGAUCACCGAAAAUGaa; and as siNC (siCtl, 4457309; Ambion, Inc.) was used. Mice were transfected with 0.25 μg/g SPL siRNA, the control siRNA or mock (siNC) by tail vain injection using Invivofectamine 3.0 (Invitrogen, Thermo Fisher Scientific).

### Assessment of liver regeneration

Proliferating cell nuclear antigen (PCNA) immunohistochemistry was performed on all collected liver specimens, using PCNA staining kit (Invitrogen, Thermo Fisher Scientific). The results were assessed as percentages or numbers of positive nuclei, using BX53 microscope and DP21 camera with ×200 objective (OLYMPUS, Japan).

### PCNA positive cell quantification

Cells with PCNA positive nucleus were counted by using an image analyzer, Fiji ImageJ (NIH Image).

### Western blotting

Proteins from liver tissues were extracted with M-PER Mammalian Protein Extraction Reagent (Thermo Fisher Scientific) plus Protease Inhibitor cocktail. The extracts were separated using Mini-PROTEAN TGX Gels (Bio-Rad, CA, U.S.A.), and blotted on to Trans-Blot, Turbo, Transfer Pack (Bio-Rad) membrane, incubated with antibodies against PCNA (1:500 dilution) and Cyclin D1 (1:200 dilution), (sc65598 and sc450, Santa Cruz Biotechnology, Texas, U.S.A.), total MAPK p42/44 and MAPK phosphorylated p42/44 MAPK (each 1:1000 dilution, 4696 and 4370, Cell Signaling Technology, Danvers, MA), SPL (1:500 dilution) and β-actin (1:2000 dilution), (ABS528 Merck Millipore and A5441 Sigma–Aldrich, St. Louis County, Missouri, U.S.A.). Immune-reactive proteins were visualized using a chemiluminescence kit (Amersham ECL Prime, GE Healthcare, Chicago, U.S.A.), and recorded using LAS-4000 image analyzer (Fuji Film, Tokyo, Japan). For the quantification of PCNA, Cyclin D1 and pERK1/2 intensities were measured by using an image analyzer, Fiji ImageJ (NIH Image).

### Quantitative real-time PCR for SPL

The total RNAs of the liver tissues were extracted using GenElute mammalian total RNA miniprep kit (Sigma–Aldrich). One microgram of purified total RNA was transcribed using a SuperScript™ First-Strand Synthesis System for RT-PCR (Roche Molecular Diagnostics, CA, U.S.A.). Quantitative real-time PCR was performed with a TaqMan Universal Master Mix (Applied Biosystems by Life Technologies, Thermo Fisher Scientific) using Verity Real Time PCR System (Applied Biosystems). SPL and internal control 18S ribosomal primers and probes (TaqMan Gene Expression Assays) were obtained from Applied Biosystems (Mm00486079 and Hs99999901). The samples were incubated for 10 min at 95°C, followed by 40 cycles at 95°C for 15 s, 60°C for 1 min. The target gene mRNA expression level was relatively quantified to ribosomal 18S using 2^−ΔΔ*C*_t_^ method (Applied Biosystems, User Bulletin No 2).

### Measurement of the S1P

For the LC-MS/MS analysis, 10 μl of homogenized liver tissues in PBS mixed with 190 μl of methanol (Wako Pure Chemical Industries, Japan) acidified with 0.1% formic acid including an internal standard. C17 base S1P (SKU-860641P, Avanti Polar Lipids, Alabama), C17 base dihydro S1P (SKU-860655P, Avanti Polar Lipids, Alabama) at 1.0 ng/ml (final concentration) were used as internal standards. The samples were sonicated for 3 min in an ultrasonic bath and centrifuged at 13000 rpm for 10 min at 4°C; then, the supernatants were collected and injected for LC-MS/MS analysis.

The LC-MS/MS analysis was performed using an HPLC and LC-8060 coupled to aquantum triple-quadrupole mass spectrometer (SHIMADZU, Japan). One microliter of each sample was injected into an InertSustain Swift C8 PEEK column (150 mm, 2.1 mm i.d., 3 μm particle size; GL Science, Japan) at a column temperature of 45°C. For the mobile phase, we used MilliQ water acidified with 0.3% formic acid (Wako Pure Chemical Industries, Japan) as solvent A and acetonitrile (LC-MS/MS grade; Wako Pure Chemical Industries, Japan) acidified with 0.3% formic acid as solvent B. Separation of the analytes was achieved using a 12-min binary gradient. After 0.5 min of an isocratic run, the proportion of solvent B was increased over 1 min from 40 to 85%, followed by 1–3 min at 85%, 3–5 min at 95%, and then equilibrated at 40% solvent B for the remaining 5–10 min. We measured the compounds in the electrospray ionization (ESI) positive ion mode, and the analytical conditions were as follows: the nebulizer gas flow was set to 3.0l/min, the drying gas flow was set to 8.0 l/min, the heating gas flow was 8.0 l/min, the interface temperature was set to 100°C, the desolvation temperature was set to 150°C, and the heat block temperature was set to 250°C.

### Statistical analysis

All data were expressed as the mean ± standard deviation (SD). Data were processed and analyzed with GraphPad Prism 8.0 software (GraphPad Software, San Diego, CA) using the unpaired *t* test, one-way ANOVA were used to analyze differences in the mRNAs levels of SPL, and the levels of S1P in tissue samples of the liver. The results were considered significant when *P*-values were <0.05.

## Results

### SPL mRNA levels were increased in the liver by PH

**Figure 1:**
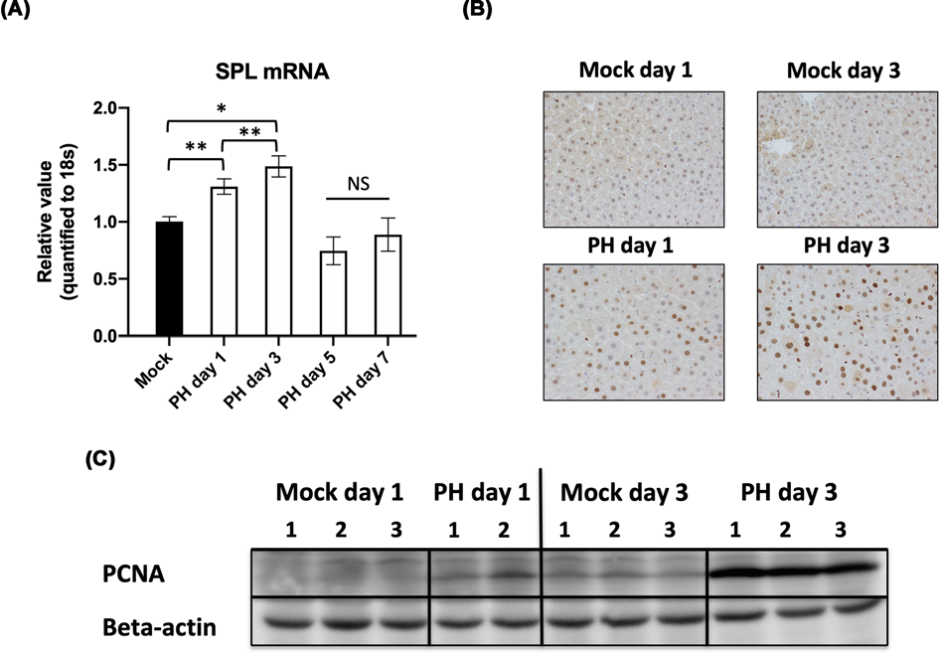
Coincided expression of the SPL with PCNA during active liver regeneration (**A**) The mRNA expression levels of the SPL in liver tissues after PH on day 1 (*n*=3), day 3 (*n*=5), day 5 (*n*=3) and day 7 (*n*=3) in comparison with mock mice (*n*=3) liver tissue. On day 3, an expression level was more enhanced than other days. (**B**) Representative IHC images of the PCNA in mock or PH liver tissues on days 1 and 3 (×200 objective). In mock liver tissues, there were no differences between days 1 and 3, however, PCNA positive hepatocytes similar to SPL increased more in liver tissues after PH on day 3 than those in day 1. (**C**) WB analysis of the PCNA. The same samples were used in (B,C) and similarly more expression of the PCNA protein observed in PH tissues on day 3. Asterisks indicate significant differences between groups as follows: *P*<0.05 *, *P*<0.01 **. NS, not significant vs mock.

In our previous study, as mentioned in the introduction we confirmed enhanced expression levels of the SPL could increase the cell proliferation rate of the HCC cells [24]. To confirm the involvement of the SPL into liver regeneration, first, we measured mRNA expression levels of the SPL in liver tissues after PH at selected time points as described in Figure 1A. The mRNA expression levels of the SPL were increased at day 1 and day 3 after PH significantly, in comparison with liver tissues of mock (sham-operated) mice. Enhanced levels of the SPL at day 3 led us to check the enzyme required for DNA synthesis [27], PCNA as a marker of the active regeneration of the PH liver. For this purpose, we performed IHC of the liver tissue slides and its representation is depicted in Figure 1B. Similar to Figure 1A, we also compared PCNA positive cells between mock mice and mice on day 1 or day 3 after PH. The data showed no changes in PCNA positive cells between day 1 and day 3 of the mock groups, but more PCNA positive cells visible on day 3 than those on day 1 of the PH group. The quantification of the PCNA positive cell numbers (data not shown) also performed and similar significant changes were observed. We confirmed these data by performing WB (Figure 1C). Considering these results, we investigated the roles of SPL in the mice on the third day after PH.

### Hepatic SPL levels were successfully suppressed by *in-vivo* siRNA

**Figure 2:**
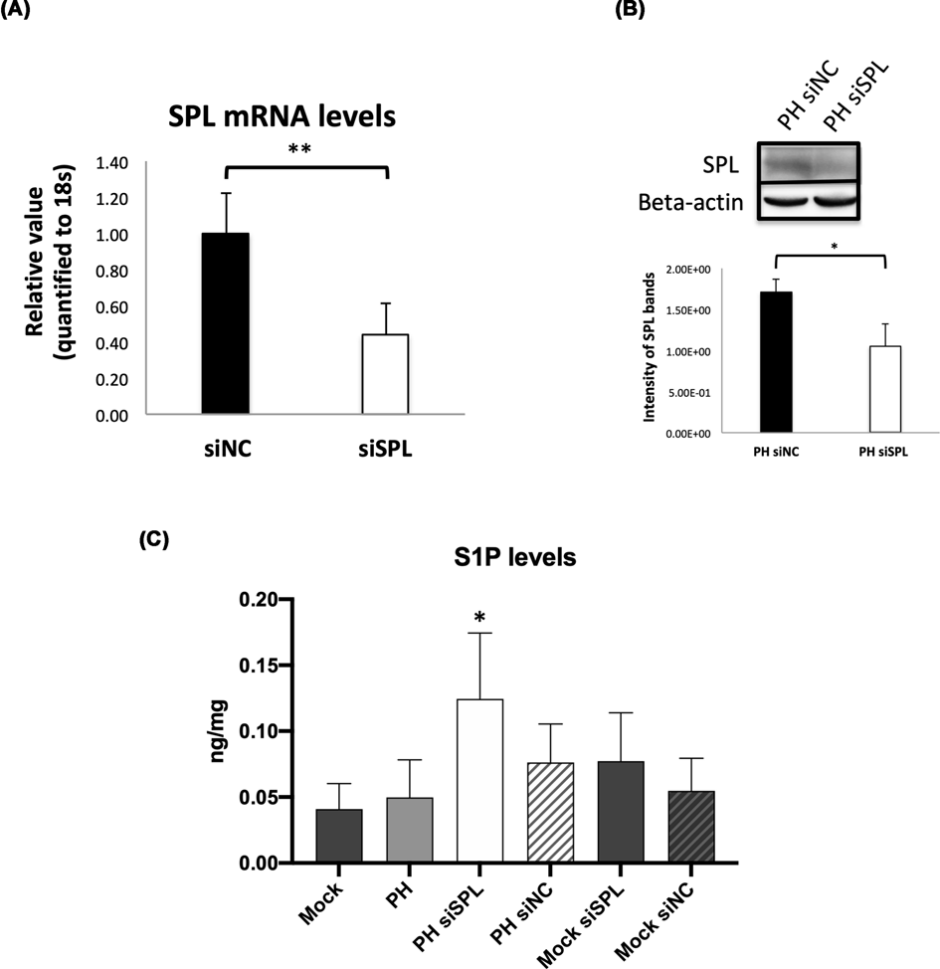
Successful silencing of the SPL in PH liver tissues by using *in-vivo* siRNA transfection (**A**) Average expression levels of the SPL in siSPL transfected mice liver tissues (*n*=10). In comparison with siNC transfected tissues (*n*=8), SPL mRNA expression levels were significantly decreased (*P*<0.01**). (**B**) Representative WB analysis data with densitometry results. Confirmation of the successful silencing in the siSPL injected PH group made by performing WB analysis. Densitometry measurement carried out with the usage of the NIH ImageJ software. (**C**) The enhanced level of the S1P in SPL inhibited liver tissues. SPL inhibition confirmed by measuring exact S1P levels in mock (*n*=4), PH (*n*=8), PH with siNC (*n*=8) or PH with siSPL (*n*=9), mock with siNC (*n*=4) or mock with siSPL (*n*=4) transfected groups. Only in PH with the siSPL transfection level of the S1P significantly increased (*P*<0.05*) due to decreased SPL, which degrades S1P irreversibly.

To investigate the involvement of the SPL and to find out its importance in liver regeneration we suppressed hepatic SPL levels, using *in-vivo* siRNA technology. As shown in Figure 2A, SPL expression levels in siSPL-injected mice were inhibited significantly in comparison with those in siNC-injected mice. We also confirmed the inhibition of the SPL in protein levels in the livers of siSPL-injected mice (Figure 2B).

Because SPL is an S1P degrading enzyme, as described in the introduction, we examined if the inhibition of SPL might result in the increase in S1P levels in mouse liver. As depicted in Figure 2C, the knockdown of the SPL led to accumulation of the S1P in liver tissues of siSPL-injected PH mice, compared with mock, PH, siNC injected PH and siNC or siSPL-injected mock mice.

### SPL inhibition led to suppressed liver regeneration

**Figure 3:**
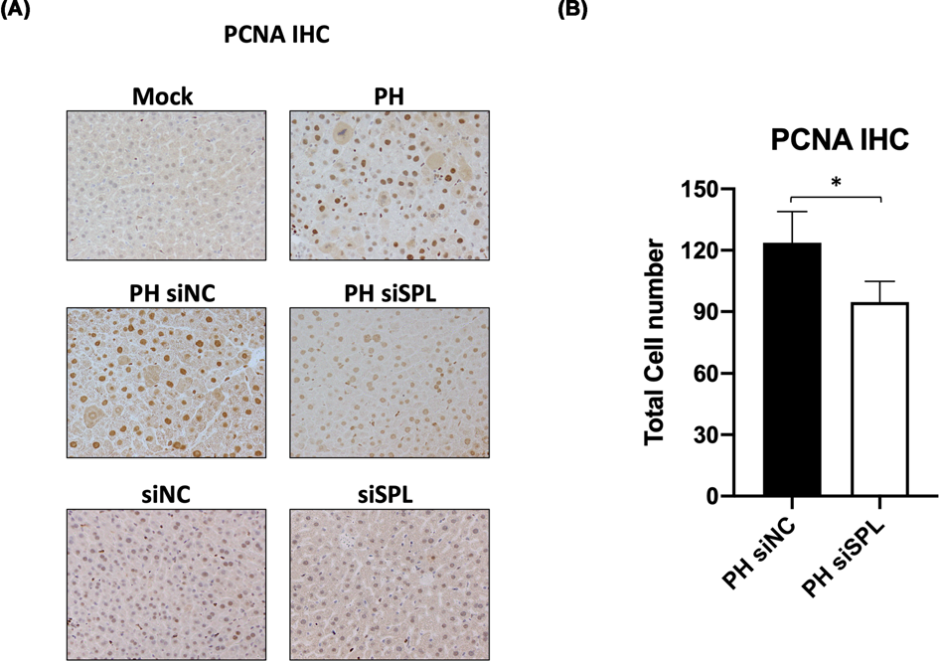
PCNA analysis shows the decreased ability of the liver regeneration in PH siSPL group (**A**) A representative IHC image of the PCNA (×200 objective). Liver tissue samples from different groups (mock, PH, PH with siNC or siSPL and only siNC or siSPL) were used for the detection of the PCNA positively stained hepatocytes. In PH performed groups, only the siSPL transfection showed decreased PCNA-stained hepatocytes. (**B**) PCNA positive hepatocytes counted in PH with siNC (*n*=8) or siSPL (*n*=10) groups. Significant differences (*P*<0.05*) observed between two groups regarding PCNA positive hepatocytes, which shown in total cell counts.

Among assays used to assess liver regeneration, we performed IHC of the PCNA. PCNA expression is induced in late G_1_, peaks in S phase and reduced thereafter [28] and it means actively proliferating cells, being mainly at S phase. As described in Figure 3A, the liver tissues of the mock and siNC or siSPL without surgery groups were weakly stained with PCNA. Liver cells with positive staining of PCNA were more observed in mice with PH only, PH with siNC treatment (Figure 3A). When we quantified, we observed that PCNA positive cell numbers were significantly fewer in siSPL-injected PH group than in siNC-injected PH groups. The suppression of the regeneration confirmed by total cell numbers (Figure 3B). These results suggest importance of the SPL in the liver regeneration.

### Status of the other proliferation markers in SPL-inhibited liver

**Figure 4:**
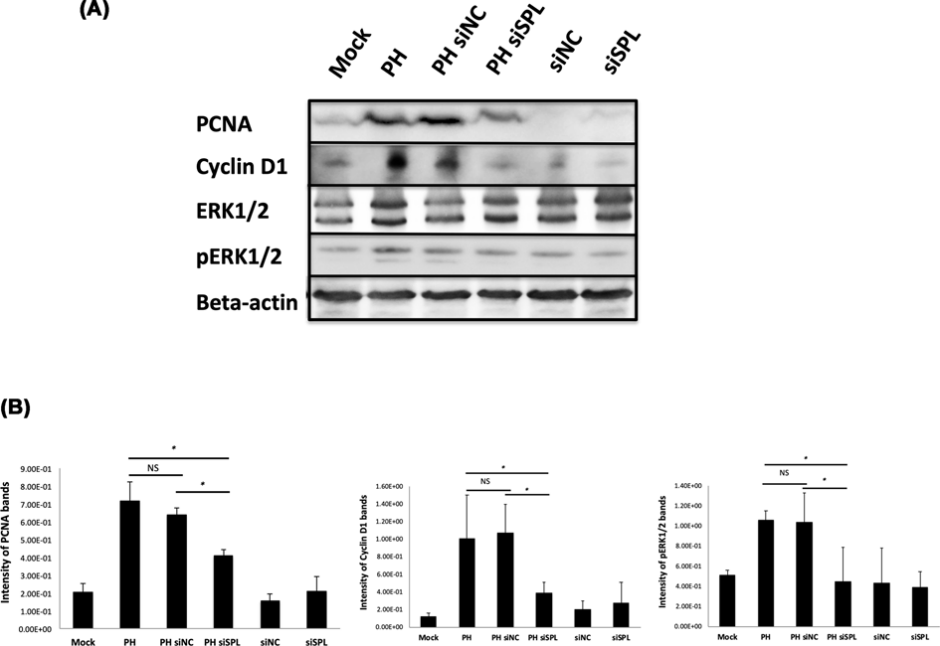
Decline of the other cell proliferation markers in PH with siSPL mice liver tissue samples (**A**) WB analysis of the cell proliferation markers including PCNA in different groups. Increased protein levels of the PCNA and Cyclin D1 together with phosphorylated ERK1/2 p42/44 MAPK observed in PH or PH with siNC injection. In PH with siSPL group expression levels of the above markers were near to other control groups where PH is not performed. (**B**) Intensities of the PCNA, Cyclin D1 and pERK1/2 protein bands (*n*=3). There were significant (*P*<0.05*) differences between PH or PH with siNC group and PH siSPL group.

Next, we analyzed some proteins with important roles in actively proliferating cells as a representative of the cell proliferation markers including PCNA. As shown in Figure 4A, Cyclin D1 protein levels of the siSPL-injected PH group livers were almost at the same level as the mock or liver tissues without PH. Cyclin D1 is known to initiate further cell cycle components and its expression patterns were similar to PCNA. Similar expression patterns were observed in phosphorylated ERK1/2 p42/44 MAPK, also known as a molecule in signaling pathway, activated in response to a diverse range of extracellular stimuli involved in many cellular programs such as cell proliferation, differentiation, motility, and death [29–31]. The intensity of the WB bands measured and quantitated (Figure 4B).

## Discussion

The present study showed the necessity of the S1P lyase (SPL) in liver regeneration. Using a PH model of the mice known to be a most reliable model to study regeneration with minimal liver injury, synchronized cell cycle, and very high reproducibility first, we confirmed the enhancement of the SPL expression due to PH on day 3 after surgery. The increased expression level of the SPL coincided with the expression of the PCNA in the same samples (Figure 1). These results gave us a hint to predict the necessity of the SPL during active liver regeneration. Similar to previous studies [21,22,24], due to suppressing the SPL by using *in vivo* siRNA led to an accumulation of the S1P observed in PH with an siSPL-injected group (Figure 2C) which may inhibit hepatocyte proliferation and following confirmation made by the significant decline of the PCNA positive cell numbers, in a group where SPL is inhibited by *in-vivo* siRNA (Figure 3). Furthermore, other proliferation markers Cyclin D1 and ERK1/2 expressions or phosphorylation also were decreased in siSPL injected PH group in comparison with PH or siNC injected PH group (Figure 4).

SPL is responsible for the ultimate step in S1P degradation as described in the introduction. Therefore, SPL may also regulate not only intracellular levels of S1P but also the amount of S1P available for extracellular export, which impacts autocrine or paracrine signaling through extracellular S1P receptors. Regarding S1P receptors, a study relating to HDL-bound S1P showed promotion of the liver regeneration after PH through its endothelial S1P_1_ receptor [10] and gave accent into the contribution of the HDL constituent ApoM. But, we previously showed possible inhibitory effect of the S1P in liver regeneration via S1P_2_ receptor [23]. Also, the products of SPL reaction may themselves influence cell proliferation by stimulating mitogenesis through S1P independent mechanism described here [32]. Recent studies also suggesting diverse roles of the S1P and the intermediate product of S1P degradation by SPL ethanolamine phosphate in the modulation of autophagy [33–35]. Considering these previous reports, SPL is necessary to support liver regeneration not only by regulating intra and extracellular levels of the S1P but also by up-regulating cellular proliferation by intermediate products from S1P degradation.

Along with the mechanism involving S1P and S1P receptors, there remain the possibilities that SPL might affect the homeostasis of lipids in a non-S1P manner. Actually, in addition to decreased regeneration, we also observed the occurrence of the fatty liver in three out of ten mice. It is already presented before that SPL deficiency disrupts lipid homeostasis in the liver by studying lipid metabolism in SPL knockout mice [36]. In addition to this another study with inhibition of SPL expression led to dysregulated processing of the transcription factor sterol regulatory element-binding protein (SREBP) [37]. Thus, SPL can be viewed as an enzyme stated between several lipid metabolic pathways.

Taken together, disruption and dysregulation of the lipid homeostasis including enhanced levels of the S1P due to inhibition of the SPL, diverse effects of the S1P through its binding receptors, S1P-independent aspects of the lipid homeostasis and modulation of autophagy by SPL products may explain the necessity of the SPL in liver regeneration. Thus, further studies to clarify the exact mechanism should be performed. In the present study, we used an *in vivo* siRNA method to suppress the expression of SPL. Theoretically, siRNA might be taken up by hepatocytes to a greater extent than other hepatic component cells. Therefore, in the present study, it remained unclear how the modulation of the SPL expression in hepatocytes might contribute to the regeneration of the whole liver tissue, which is composed of many kinds of cells. Besides these, in further studies, the effect of the siSPL in other tissues and their impact on liver regeneration in a similar condition with longer period of time should be considered.

To our knowledge, the current study is the first report to show the importance of the SPL in liver regeneration by using a murine PH model. SPL might be a potential target to facilitate liver regeneration.

## Abbreviations

HCC: hepatocellular carcinoma
HDL: high density lipoprotein
IHC: immunohistochemistry
i.d.: inside diameter
PCNA: proliferating cell nuclear antigen
PH: partial hepatectomy
SK1: sphingosine kinase 1
SPP: sphingosine 1-phosphate phosphatase
S1P: sphingosine 1-phosphate
WB: western blotting
